# Vancomycin Resistance Is Overcome by Conjugation of Polycationic Peptides

**DOI:** 10.1002/anie.202002727

**Published:** 2020-04-21

**Authors:** Florian Umstätter, Cornelius Domhan, Tobias Hertlein, Knut Ohlsen, Eric Mühlberg, Christian Kleist, Stefan Zimmermann, Barbro Beijer, Karel D. Klika, Uwe Haberkorn, Walter Mier, Philipp Uhl

**Affiliations:** ^1^ Department of Nuclear Medicine Heidelberg University Hospital Im Neuenheimer Feld 400 69120 Heidelberg Germany; ^2^ Institute of Pharmacy and Molecular Biotechnology Heidelberg University Germany; ^3^ Institute for Molecular Infection Biology (IMIB) University of Würzburg Germany; ^4^ Medical Microbiology and Hygiene Heidelberg University Hospital Germany; ^5^ German Cancer Research Center (DKFZ) NMR Spectroscopy Analysis Unit Germany; ^6^ Department of Nuclear Medicine Heidelberg University Hospital Germany; ^7^ Clinical Cooperation Unit Nuclear Medicine German Cancer Research Center (DKFZ) Germany; ^8^ Translational Lung Research Center Heidelberg (TLRC) German Center for Lung Research (DZL) Germany

**Keywords:** antibiotics, bacterial resistance, glycopeptide antibiotics, peptide conjugates, vancomycin

## Abstract

Multidrug‐resistant bacteria represent one of the biggest challenges facing modern medicine. The increasing prevalence of glycopeptide resistance compromises the efficacy of vancomycin, for a long time considered as the last resort for the treatment of resistant bacteria. To reestablish its activity, polycationic peptides were conjugated to vancomycin. By site‐specific conjugation, derivatives that bear the peptide moiety at four different sites of the antibiotic were synthesized. The most potent compounds exhibited an approximately 1000‐fold increased antimicrobial activity and were able to overcome the most important types of vancomycin resistance. Additional blocking experiments using d‐Ala‐d‐Ala revealed a mode of action beyond inhibition of cell‐wall formation. The antimicrobial potential of the lead candidate FU002 for bacterial infection treatments could be demonstrated in an in vivo study. Molecular imaging and biodistribution studies revealed that conjugation engenders superior pharmacokinetics.

## Graphical abstract

**Resistance is futile**: By site‐specific conjugation polycationic peptides were conjugated to vancomycin. Derivatives that bear the peptide moiety at four different sites of the antibiotic were synthesized. The most potent compounds exhibited an approximately 1000‐fold increased antimicrobial activity and were able to overcome the most important types of vancomycin resistance.

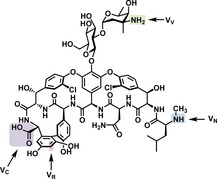

At first glance, the treatment of bacterial infections seems to be a straightforward task. As the cellular structures of prokaryotes differ greatly from those of the human host, they can be readily targeted by drugs, and nature has conjured up a multitude of antimicrobials to do just that.^1^ Owing to their perfection by eternal natural evolution, antimicrobials can attain extraordinary performance in interfering with various bacterial mechanisms. The few compounds that combine this functionality with suitable pharmacokinetic properties, thereby allowing medicinal application, represent the main source for antibiotic drugs.^1^ This repertoire of drugs is further complemented by some classes of synthetic antibiotics, such as quinolones and oxazolidinones.^2^ For a long time, this pool of drugs seemed to be the preeminent solution for the treatment of bacterial infections. Filled with self‐assured certainty, mankind embarked on a carefree and extensive use of antibiotics^3^ with the consequence that over the course of time, the reservoir of effective drugs has decreased as a result of the progressive development of bacterial resistance.^4^ Unfortunately, the pharmaceutical industry has largely stopped antibiotic development, thus putting the onus on scientists to provide new potent therapies.^5^ To prevent the imminent post‐antibiotic era, major efforts must therefore be made to maintain the availability of effective antibiotic therapies. The preferred strategy for antibiotic development should be the optimization of seasoned compounds to break the development of resistance. This new strategic direction can either consist of attacking bacteria using multiple mechanisms and/or reinforcement of the mode of action. Herein, we demonstrate the usefulness of this new strategy for the glycopeptide antibiotic vancomycin.

Vancomycin is the critical and decisive antibiotic for the treatment of multidrug‐resistant infections caused by Gram‐positive bacteria.^6^ The highly cross‐linked heptapeptide core structure of vancomycin minimizes the loss of conformational entropy upon binding, leading to micromolar binding affinities. Predominantly enthalpy‐driven interactions are usually restricted to highly structured proteins, for instance avidin, the catalytic domains of enzymes or the binding site in the Fv region of antibodies. Vancomycin therefore represents a rare example of a small molecule with the capability of high‐affinity binding. Owing to one of the tightest bindings known for low‐molecular weight organic compounds, vancomycin is capable of neutralizing the peptide moiety of the cell wall precursor lipid II.^7^ Ever since its discovery in 1958, vancomycin seemed to be a drug devoid of the threat of resistance development. Unfortunately, since 1988, various vancomycin‐resistant strains have been described.^8^ Vancomycin‐resistant enterococci, as well as staphylococci, are currently classified as “high priority” pathogens by the WHO.^9^ To circumvent inhibition of cell‐wall formation, resistant Gram‐positive bacteria have developed mechanisms to modify the d‐Ala‐d‐Ala motif of the pentapeptide of lipid II, the target structure of vancomycin. The most important resistant strains substitute the C‐terminal d‐Ala by its oxa analogue lactic acid or serine.^10^ Herein we develop a strategy to overcome bacterial resistance by the synthesis of vancomycin‐polycationic peptide conjugates. Polycationic peptides have also been assessed as potential delivery vehicles, for example, for the delivery of proteins^11^ and nanobodies^12^ into cells. Furthermore, in a recently published study, surface modification of nanoparticles with cyclic polycationic peptides highly increased the oral uptake of the peptide drug liraglutide.^13^

In our study, the short polycationic peptides were obtained by solid‐phase peptide synthesis. Hexa‐arginine was found to be the most efficient peptide. This peptide was extended by a cysteine residue to enable conjugation via a heterobifunctional cross linker (Scheme 1). Using site‐specific reactions, vancomycin could be modified at four different sites, named V_N_, V_R_, V_C_, and V_V_ (Scheme 2), resulting, for example, in the compounds FU002, FU007, FU008 or FU013 (Scheme 2 and Table S1 in the Supporting Information).

**Scheme 1:**
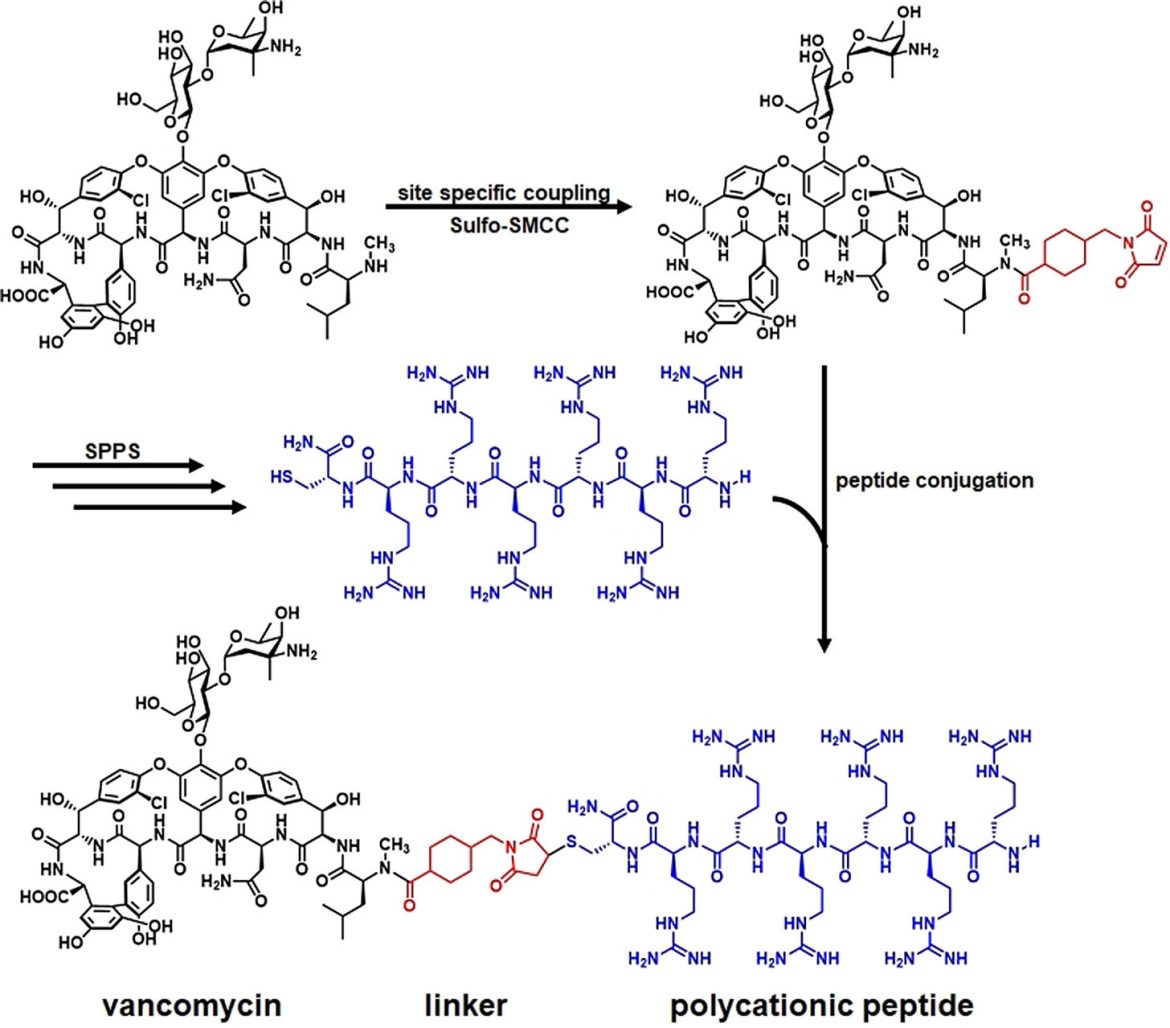
Synthesis of the peptide conjugates, representatively shown for the lead candidate FU002. In the first step, vancomycin is coupled to a heterobifunctional cross linker SMCC (succinimidyl 4‐(*N*‐maleimidomethyl)cyclohexane‐1‐carboxylate). Michael addition of a cysteine residue to the maleimide functionality leads to a stable peptide‐antibiotic conjugate.

**Scheme 2:**
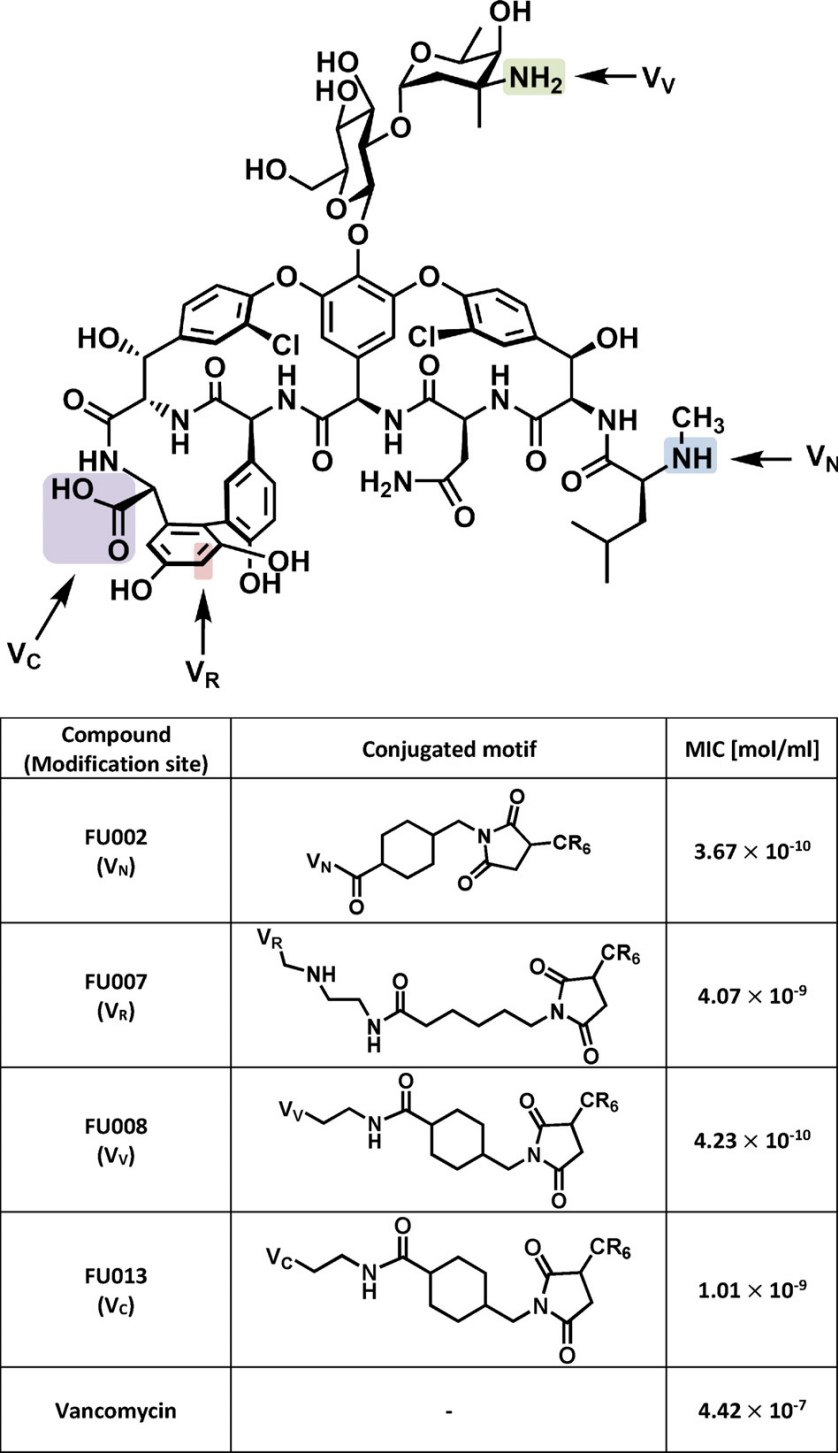
The structure of vancomycin with the coupling positions indicated for V_N_, V_R_, V_C_, and V_V_. The table lists the antimicrobial activities of selected compounds on *E. faecium* UL602570*, a clinical isolate.

The site selectivity of the conjugation to the amine functionalities, that is, V_N_ versus V_V_, was confirmed by NMR spectroscopy. To unambiguously verify the specificity of conjugation to the preferred coupling site V_N_ for the lead candidate, FU002 was cleaved under acidic conditions using trifluoroacetic acid. The analysis of the deglycosylated reaction product clearly showed the vancomycin‐peptide conjugate devoid of the saccharide moiety thus indicating that coupling had indeed occurred at the preferred site (see Figure S4). The method of choice for the evaluation of the antimicrobial potential of compounds remains the broth microdilution method. The clinical breakpoint for vancomycin resistance on enterococci is defined as a concentration >4 mg L^−1^ by the European Committee on Antimicrobial Susceptibility Testing (EUCAST). The MIC (minimum inhibitory concentration) values of the lead compound FU002 determined in this study (notably with all values below 4 mg L^−1^) revealed that the most important types of vancomycin resistance (vanA, vanB and vanC) could be overcome (see Figure 1 A), suggesting the use of FU002 for the treatment of all common vancomycin‐resistant strains. FU002 also shows MIC values comparable to those of vancomycin for sensitive strains (Figure S1). The superior performance of the derivatives over vancomycin towards vancomycin‐resistant strains suggests the presence of a second mode of action in addition to the binding to lipid II pentapeptide. Further analysis of the binding to the cell‐wall precursors could help to verify this assumption. If the derivatives follow a dual mechanism, this would be a basis for resilience against the development of resistance. First insights could be obtained in preliminary resistance induction studies on the vancomycin‐sensitive *Staphylococcus aureus* strain MRSA USA300 LAC as no resistance development was observed after eight passages in increasing concentrations of FU002 (Figure S8).

**Figure 1:**
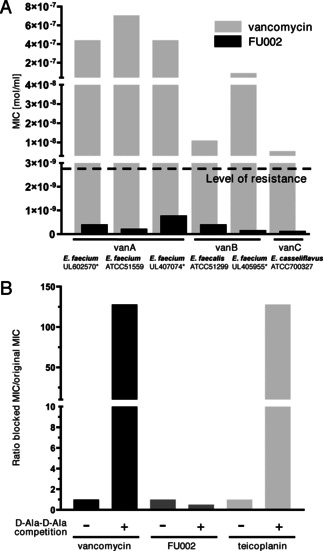
Antibacterial activity of vancomycin and its derivative FU002. A) FU002 vastly outperforms vancomycin regardless of the type of vancomycin resistance (*=clinical isolate). B) Blocking experiments with *Nα*,*Nϵ*‐diacetyl‐Lys‐ d‐Ala‐d‐Ala binding motif on *Staphylococcus aureus* NCTC 10442. The d‐Ala‐d‐Ala binding motif suppresses the antibiotic activity of vancomycin and the related glycopeptide antibiotic teicoplanin. In contrast, FU002 preserves its activity, indicating a mode of action that is not cell‐wall precursor binding.

Although the exact mode of action cannot be provided so far, the number of possibilities can be narrowed down: As the conjugate shows neither any relevant activity on Gram‐negative strains (Table S2) nor significant hemolytic activity a general mechanism mainly based on lysis of cells can be excluded. Moreover, synergistic effects on the bacterial cell wall are not likely owing to the lack of activity of co‐administered vancomycin and the peptide moiety (Table S3). Targeting solely the d‐Ala‐d‐Ala motif by the vancomycin moiety of the conjugates could be excluded as FU002 shows no relevant change in MIC values in blocking experiments (Figure 1 B). Besides, a high antimicrobial potential is not the only requirement for a compound to serve as an ideal antibiotic as frequently the potential of a potent substance cannot be exploited due to adverse reactions, for example, high cytotoxic activity.

Fortunately, however, FU002 shows promising non‐toxic characteristics as demonstrated by the absence of hemolysis of human blood cells and good cytocompatibility as demonstrated by in vitro cytotoxicity studies using the XTT assay. This tolerability could be demonstrated for all relevant cell lines (blood, kidney, and liver cells) at all tested concentrations of FU002 (see Figure S5 B).

For therapeutic antimicrobial substances to be of value, their antimicrobial activity must be combined with preferred pharmacokinetics. Unmodified vancomycin, for example, is known to be predominantly excreted via the kidneys. However, in patients with impaired renal function the risk of associated nephrotoxicity is high, which is the major limitation for the application of vancomycin under these conditions.^14^ Encouragingly, our conjugates, as a result of the conjugation of a peptide moiety, are partially directed to the hepatobiliary excretion route, thereby extending their biodistribution characteristics. The modified elimination profile of FU002 was tracked by molecular imaging and biodistribution studies to reveal its improved pharmacokinetic profile.

For molecular imaging by scintigraphy or positron‐emission tomography (PET), vancomycin and the lead candidate FU002 were radiolabeled with either ^125^I or ^124^I for the studies in female Wistar rats (see Figure 2 and Figure S7). FU002 remains in the liver for several hours, which can be highly advantageous for the treatment of liver infections while, in contrast, vancomycin, with its fast renal clearance, is not suitable for such applications. Of note, these changes in the biodistribution and elimination routes relative to vancomycin raise the hopeful prospect that even high doses of the new conjugates can be tolerated by humans, thus enabling therapy options with respect to severe and complicated infections arising from multidrug‐resistant bacteria. Previously, several strategies to obtain vancomycin derivatives with high potency against vanA‐resistant bacteria have been published.^15^ However, even the compounds with the highest antibacterial potential were not suitable for in vivo application owing to their structural instability in plasma^16^ or the requirement of complex organic synthesis that exceed the efforts justifiable for broad application purposes.^17^, ^18^, ^19^ In contrast, the conjugation strategy used herein enabled the straightforward and reproducible synthesis of a variety of vancomycin conjugates. As the synthetic procedure can readily be scaled up, it is the basis for the synthesis of sufficient quantities for preclinical and, potentially, clinical application.

**Figure 2:**
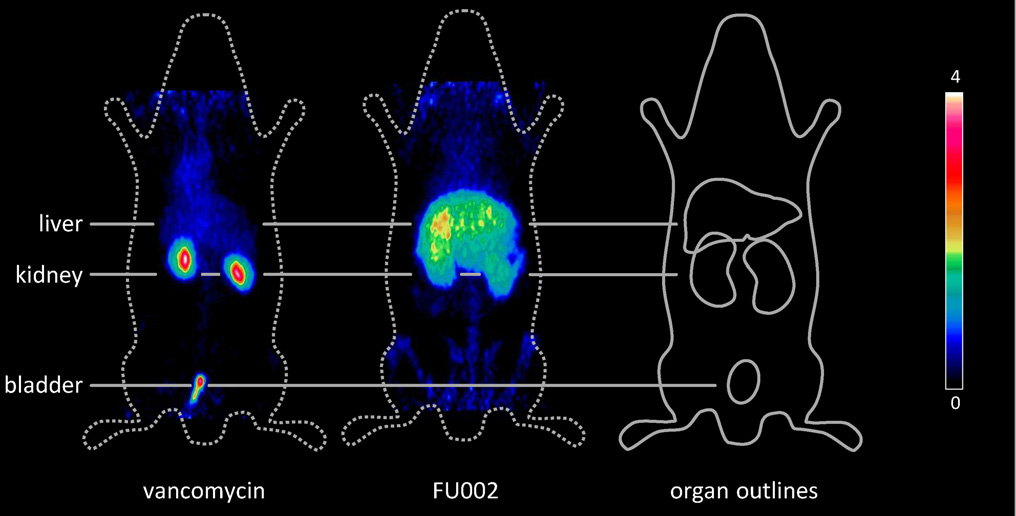
Molecular imaging to visualize the pharmacokinetics of vancomycin and FU002. Shown is the PET imaging of ^124^I‐labeled vancomycin and its ^124^I‐labeled derivative ^8^Tyr‐FU002 at 10 min post injection. While vancomycin shows renal excretion, ^124^I‐^8^Tyr‐FU002 has a broader distribution profile.

FU002 was chosen as the lead candidate because of its high antimicrobial potential overcoming all types of vancomycin resistance. Such a high antimicrobial potential for a modified vancomycin derivative has not been reported before with respect to vanA‐, vanB‐ and vanC‐resistant bacteria. Additionally, the antimicrobial potential of FU002 for bacterial infection treatments could be confirmed in an in vivo efficacy study. In *Staphylococcus aureus* USA300 LAC infected mice, FU002 was shown to be effective, as demonstrated by a stable body weight despite the infection and the significant reduction of the colony forming units (Figure 3 A,B).

**Figure 3:**
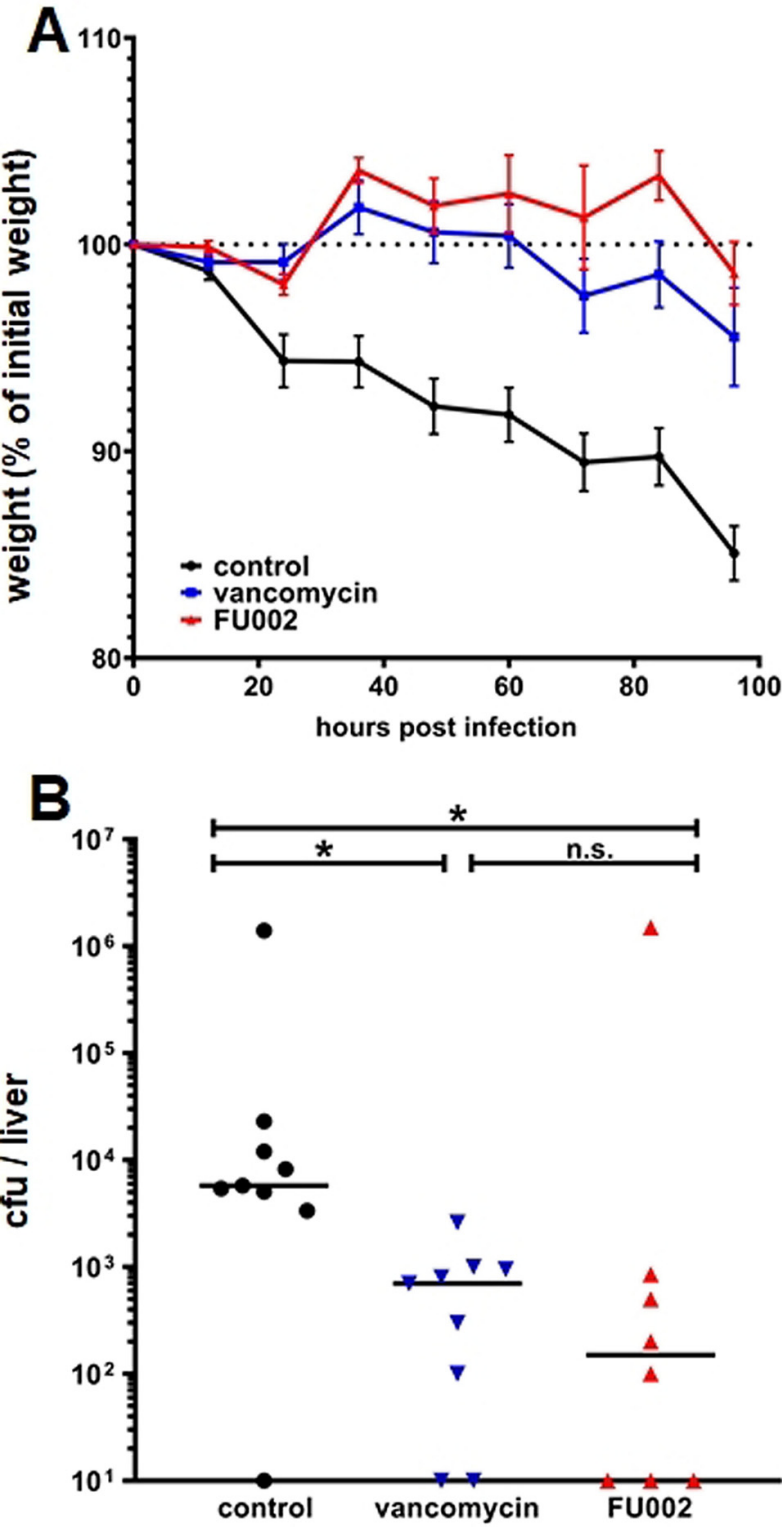
In vivo efficacy model of FU002 and its progenitor vancomycin in *Staphylococcus aureus* USA300 LAC (MRSA) infected mice. As negative control, 0.9 % NaCl was used. A) No infection‐related body weight loss can be observed for FU002. It therefore shows comparable good treatment efficacy compared to vancomycin in a vancomycin sensitive infection model. B) The in vivo activity of FU002 could be confirmed by a significant reduction of bacterial burden, as shown for the colony forming units (CFU) in the liver (*p < 0.05).

In summary, vancomycin conjugation to polycationic peptides represents a promising tool and convincingly illustrates a new strategy for the development of highly potent and non‐toxic antimicrobial substances by structural modification of already approved drugs to combat one of the biggest challenges in today's medicine—multidrug‐resistant bacteria. Furthermore, peptide conjugation allows controlling the biodistribution and excretion profile of novel compounds making them more flexible for their broad application against a variety of multidrug‐resistant bacteria.

## Conflict of interest

The authors declare no conflict of interest.

## Supporting information

As a service to our authors and readers, this journal provides supporting information supplied by the authors. Such materials are peer reviewed and may be re‐organized for online delivery, but are not copy‐edited or typeset. Technical support issues arising from supporting information (other than missing files) should be addressed to the authors.

Supplementary
