# Supplementary material for: Vancomycin Resistance Is Overcome by Conjugation of Polycationic Peptides
Source: Angew Chem Int Ed Engl. 2020 Apr 21;59(23):8823–7. doi: 10.1002/anie.202002727 (PMC7323874; doi:10.1002/anie.202002727)
Supplement: Supplementary file 1 — Supplementary [file ANIE-59-8823-s001.pdf]

Angewandte Chemie
Eine Zeitschrift der Gesellschaft Deutscher Chemiker

Supporting Information

Vancomycin Resistance is Overcome by Conjugation of Polycationic Peptides

*Florian Umstätter, Cornelius Domhan, Tobias Hertlein, Knut Ohlsen, Eric Mühlberg, Christian Kleist, Stefan Zimmermann, Barbro Beijer, Karel D. Klika, Uwe Haberkorn, Walter Mier, and Philipp Uhl**

anie_202002727_sm_miscellaneous_information.pdf

Supplementary data:

Materials

For peptide synthesis, all Fmoc-L amino acids were purchased from Orpegen Peptide Chemicals GmbH, Heidelberg, Germany. Fmoc-D-tyrosine as well as the rink amide resin were obtained by Iris Biotech GmbH, Marktredwitz, Germany. Vancomycin.HCl for synthesis of conjugates was derived from Noridem Enterprises Limited, Nicosia, Cyprus and Hikma Pharma GmbH, Planegg, Germany. Ethylene diamine for derivatization of vancomycin was obtained from Lancaster, Mühlheim am Main, Germany. Sulfo-SMCC for crosslinking was purchased from Carbosynth, Limited, Compton, Berkshire, United Kingdom as well as Iris Biotech GmbH, Marktredwitz, Germany. 6-maleimidohexanoic acid N-hydroxysuccinimide ester for the V_R position was purchased from Iris Biotech GmbH, Marktredwitz, Germany. In general, purification was performed by preparative HPLC using a LaPrep P 110 (VWR International, Karlsruhe, Germany) HPLC system equipped with a Reprosil™ Gold 120 C-18 column (4 µm, 150 x 20 mm; Dr. Maisch HPLC GmbH, Ammerbuch, Germany). Analysis was performed by LC/MS using a Thermo Scientific Exactive mass spectrometer with a gradient of water and acetonitrile containing 0.05% TFA and a flow rate of 200 µl/min for HPLC. Vancomycin.HCl (potency 99.8%) as control in microdilution assays was purchased from Sigma-Aldrich Chemie GmbH, München and teicoplanin (Targocid®) from Sanofi, Frankfurt, Germany. Nα,Nε-Diacetyl-Lys-D-Ala-D-Ala for blocking experiments was purchased from BACHEM, Bubendorf, Switzerland. For adjustment of the cell number in the microdilution assay a McFarland-counter DensiCHEK® plus, bioMerieux, Marcy-l'Étoile, France was used. Growth medium Mueller-Hinton-Broth II (cation-adjusted) was purchased from Sigma Aldrich. For microdilution assays polypropylene, U-bottom 96-well plates were purchased from Greiner Bio-One GmbH, Frickenhausen, Germany. All clinical isolates were obtained from the Institute for Medical Microbiology and Hygiene, Heidelberg University Hospital, Heidelberg, Germany. All other reference strains were supplied by the Department of Infectious Diseases, Medical Microbiology and Hygiene, Heidelberg University, Heidelberg, Germany. *Staphylococcus aureus* USA300 LAC was supplied by the Institute for Molecular Infection Biology, Würzburg University, Würzburg, Germany. For absorbance measurements in hemolysis and cytotoxicity studies an Infinite M200 PRO microplate reader, Tecan Trading, Maennedorf, Switzerland was used. 2,3-Bis-(-2-methoxy-4-nitro-5-sulfophenyl)-2*H*-tetrazolium-5-carboxanilide salt containing the activation reagent phenazine methosulfate for cytotoxicity studies was purchased from Applichem, Darmstadt, Germany. Costar, Corning, Tewksbury, MA, USA 96-well plates were used for seeding and the assay itself. For the hemolysis assay, v-bottom and flat-bottom polystyrene plates were purchased from Greiner Bio-One International GmbH, Kremsmünster, Austria. TRITON® X-100 used as control for hemolysis was from Sigma Aldrich, Steinheim, Germany. For animal studies, rats were grown by Janvier Labs, Le Genest-Saint Isle, France. ^{124}I was purchased from PerkinElmer, Boston, USA. ^{125}I were purchased from Hartmann Analytic GmbH, Braunschweig, Germany. For scintigraphical images, a γ-camera (Gamma Imager, Biospace Lab, Paris, France) and for PET imaging, an Inveon small animal PET scanner (Siemens, Erlangen, Germany), were used. The remaining radioactivity in the specific organs for biodistribution studies was measured with a Cobra Auto γ-Counter, Packard BioScience Co., Meriden, CT, USA.

Experimental section

Peptide synthesis

All peptides were synthesized by solid phase peptide synthesis using the Fmoc-strategy as described previously.[1] Therefore, a rink amide resin was preloaded with 0.67 mmol/g of cysteine for coupling to the C-terminus of the peptide. The further amino acids were coupled in standard strategy using an Applied Biosystems 433A synthesizer with the HBTU activation strategy. After coupling was completed, the resin was washed with dichloromethane and diethyl ether and dried in vacuum. The final cleavage of the peptide was performed in TFA/H_2O/TIS (90/5/5) for at least two hours. The cleaved peptide was precipitated in diethyl ether and dried in vacuum. Analytics were performed by HPLC with a linear gradient of 0.1% TFA in water (eluent A) to 0.1% TFA in acetonitrile (eluent B) within 5 min; flow rate 2 ml/min; UV absorbance λ = 214 nm and mass spectrometry. Purification of the peptides was performed by preparative HPLC. The purity of the final product was determined by LC/MS.

Synthesis of lead compound FU002 (V_N-position)

For the synthesis of lead candidate FU002, vancomycin hydrochloride was solved in PBS (pH 8.16). If pH was below, it was readjusted to pH 8.16. Afterwards, 0.5 eq of the bifunctional linker Sulfo-SMCC were solved in DMSO. Solutions were merged, pH was checked again and the solution was shaken at room temperature for about three hours. Reaction was controlled by HPLC analysis. After the reaction was completed, the mixture was lyophilized, resolved in water and purified by preparative HPLC using an acetonitrile/water gradient. Analysis was performed by LC/MS.
After purification, the vancomycin-linker conjugate was solved in PBS (pH 5.5) while the peptide (R6C) was solved in DMSO. Both solutions were merged and shaken at room temperature for about two hours. The reaction was controlled by HPLC. Purification and analysis were performed as described previously.

Synthesis of V_V-ethylene diamine derivatives

For the synthesis of the V_V-position, vancomycin was modified with an ethylene diamine. Therefore, vancomycin hydrochloride and N-(9-fluorethoxycarbonyl) glycinal was reacted as described before by Long et al.[2] and purified by preparative HPLC. The resulting product was deprotected with quinuclidine and used without further purification.
For the coupling of the bifunctional SMCC-linker, the ethylene diamine extended vancomycin was solved in PBS (pH 8.16) and the solution was mixed with Sulfo-SMCC in DMSO stock solution. After 30 min, the product was purified by preparative HPLC.
The coupling of the peptide was performed similar to the lead candidate FU002.

Synthesis of the V_C-ethylene diamine derivative

For addressing the carboxylic acid of vancomycin, vancomycin hydrochloride was dissolved in DMSO (0.1 mmol/ml) and 3 eq N-Ethyl-N-(propan-2-yl)propan-2-amine (DIPEA) were added. To this mixture ethylene diamine and a solution of Benzotriazol-1-ol (HOBt) and PyAOP ((7-Azabenzotriazol-1-yloxy)tripyrrolidinophosphonium hexafluorophosphate) in DMF was added. After two hours, the mixture was purified by preparative HPLC and the product was analysed by LC/MS and lyophilized afterwards.
For the SMCC coupling, the V_C-ethylene diamine derivative was solved in PBS (pH 8.16) and Sulfo-SMCC in DMSO solution was added. HPLC showed a 70% reaction after 30 min. The purification was performed as described above. The product was lyophilized and analysed by LC/MS. The peptide coupling was performed as described previously.

Synthesis of the V_R-ethylene diamine derivative

Synthesis of the V_R-position was performed with vancomycin hydrochloride, formaldehyde and ethylene diamine according to Long et al.[2] and purified by HPLC. A solution of this product (0.03 mol/l) was mixed with 6-maleimidohexanoic acid N-hydroxysuccinimide ester, stirred and purified by HPLC after two days.

Structural analysis of lead compound FU002 via deglycosylation

For the structural analysis, the test substance was dissolved in water (0.02 mol/l) and incubated in TFA (trifluoroacetic acid) overnight. TFA was removed in vacuum and the remaining substance was dissolved in water/acetonitrile (1:1). Analytical analysis was performed by HPLC and LC/MS.

Structure analysis of lead compound FU002 by NMR

NMR spectra were acquired using a Bruker Avance II NMR spectrometer equipped with a 5-mm, inverse-configuration probe with triple-axis gradient capability at a field strength of 14.1 T operating at 600 MHz for ^{1}H nuclei in *d$_6$*-DMSO at 298 K. Pulse widths were calibrated following the described protocol.[3] The chemical shifts of ^{1}H nuclei are reported relative to TMS (δ = 0 ppm) using the solvent signal as a secondary internal reference ($\delta_{CHD2SOCD3}$ = 2.50 ppm). General NMR experimental and acquisition details for 1D ^{1}H and selective 1D NOESY (τ_m, 0.3 s) and standard, gradient-selected 2D COSY and ^{1}H{^{13}C}-HSQC have been previously described[4-6] for routine spectral assignment and structural analysis.

Structural determination and characterization of the conjugates

The attachment site of the sidechain by SMCC was confirmed by 1D NOESY and changes in the chemical shifts in comparison with vancomycin.[7] The proton and carbon chemical shifts taken from HSQC of the amino sugar in SMCC do not differ from those in vancomycin whereas the shifts in the isobutyl chain are significantly shifted. The proton chemical shifts also differ in a phenyl group that can be only close to the isobutyl chain and not the vancomycin sugar and for which an NOE between protons in the introduced moiety and a proton in that phenyl group can be observed.

Antimicrobial testing – Microdilution

Antimicrobial testing was performed according to CLSI and EUCAST guidelines in microdilution.[8-10] Therefore, all tested substances as well as controls were dissolved in water in different concentrations and serially diluted in MHB II (cation adjusted) on the 96-well plate. A logarithmic growing, overnight culture of the respective bacterial strain was used and adjusted to a McFarland corresponding to 1×10^8 cfu/ml. Afterwards the bacterial suspension was diluted to 1×10^6 cfu/ml and loaded on the 96-well polypropylene plates, resulting in a final bacterial concentration of 5×10^5 cfu/ml. After an incubation at 35 $\pm$ 1 °C for 18-20 hours (re-controlled after 24 hours) the MIC was determined as the lowest concentration without visible growth as described previously.[11] For direct comparison of the tested substances, all results were calculated in mg/ml and mol/ml afterwards.
For blocking experiments with NA,NE-DIACETYL-LYS-D-ALA-D-ALA the tested substances were incubated directly on the dilution plate with at least 100-fold excess of Nα,Nε-Diacetyl-Lys-D-Ala-D-Ala for a minimum of 1 hour at room temperature. Afterwards, the procedure was performed as described above. As further control substance, the glycopeptide antibiotic teicoplanin was evaluated.

Resistance development study

Resistance development in *S. aureus* was determined by passaging bacteria in increasing amounts of vancomycin, FU002 or, as control, fusidic acid. In principle, tubes with twofold dilutions of the compounds in 1 ml Muller Hinton broth were inoculated 1:100 with bacterial suspension. The tubes were then incubated for 24 h at 37 °C under shaking. After each passage, the tube with the highest compound concentration showing bacterial growth was used to inoculate the tubes of the next passage (1:100). The tube with the lowest compound concentration showing no growth was defined as the passage MIC. Passaging was stopped after 8 passages.

Cytotoxicity studies

For the validation of possible side effects provoked by the conjugates, the cytotoxic effect of the lead candidate FU002 on human cells was assessed in a colorimetric viability assay. Different human cell types were used including kidney and liver cell lines as well as freshly isolated peripheral blood cells. Cells were seeded into 96-well microtiter plates at 2×10^4 cells/well and cultured overnight (18-24 h) in a

humidified atmosphere at 37 °C and 5% CO_2. Then, the cells were exposed to the conjugate as well as the comparator substance vancomycin at final concentrations of 1 up to 64 µg/ml. After 24 h of incubation, the XTT reagent 2,3-bis-(-2-methoxy-4-nitro-5-sulfophenyl)-2*H*-tetrazolium-5-carboxanilide salt containing the activation reagent phenazine methosulfate was added. The enzyme succinate dehydrogenase of the mitochondria in living cells is capable to reduce the tetrazolium substrate XTT resulting in orange colored formazan compounds. The intensity of the color change of the cell culture medium is proportional to the number of viable, metabolically active cells. Thus, the adsorption was subsequently measured with an Infinite M200 PRO microplate reader at 470 nm and 670 nm (reference). The percentage of viable cells was determined in relation to untreated cultured cell controls (= 100% viability).

Hemolysis

For hemolysis studies, blood from four different, fasted men were taken to determine the hemoglobin release. Erythrocytes were purified centrifuging blood at 2500 rpm for 3 min. The remaining pellet was washed with PBS and centrifuged again. This process was repeated until the supernatant remained clear.
For the hemolysis assay, v-bottom plates were used. FU002 was tested in duplicates in three different assays. As positive control, a 1% Triton® in 0.9%-NaCl solution was used.
FU002 was serial diluted on the plate, resulting in 50 µl of a final concentration of 640 µg/ml to 1.25 µg/ml. Every well contained 50 µl of the purified erythrocytes. As positive control 50 µl Triton® X-100 (1% in 0.9%-NaCl solution) were used. The plate was incubated for 120 min at 37 °C. Afterwards, 50 µl PBS were added in each well and the plate was centrifuged for 2 min at 4000 rpm. 50 µl of the supernatant were transferred to a flat-bottom plate and absorption was measured at 554 nm.

Hemolysis was calculated as followed:

$$Hemolysis\ [\%] = 100 \times \frac{A_{FU002} - A_{blank}}{A_{Triton} - A_{blank}}$$

Biodistribution and pharmacokinetic studies

The biodistribution and pharmacokinetic studies were performed with radiolabeled ($^{124}I/^{125}I$) compounds. For this radiolabeling strategy, a D-tyrosine-modified derivative was synthesized, which enabled the nucleophilic substitution. The D-tyrosine was located at the N-terminus of the respective peptide. Vancomycin was radiolabeled without any further structural modification. For radiolabeling, a 1 mM stock solution of phosphate buffer/dimethyl sulfoxide was prepared as described previously by Uhl et al.[12] The required amount of radioactive iodine-124 (^{124}I) or rather iodine-125 (^{125}I) was added to a mixture of 25 µl of the stock solution and 25 µl of a phosphate buffer (0.25 mM; pH 7.5). Radiolabeling was performed using the chloramine T method as previously described by Crim et al.[13] The labeled compound was purified by semi-preparative HPLC. Analysis was done by radio-HPLC (Agilent 1100 series) using a Chromolith® Performance RP-18e, 100–3 mm column applying a linear gradient of 0.1% TFA in water (eluent A) to 0.1% TFA in acetonitrile (eluent B) within 5 min; flow rate 2 ml/min; UV absorbance λ = 214 nm; γ-detection. The animal trials were approved by the Animal Care and Use committees at the Regierungspräsidium Karlsruhe, Germany. Adult, female, Wistar rats (200 – 250 g) were purchased from Janvier Labs (Le Genest-Saint-Isle, France). The animals were anaesthetized by isoflurane inhalation and appropriate amounts of the $^{124}I/^{125}I$ labelled peptides, dissolved in 100 µl of 0.9% NaCl were injected into the tail vein. For PET-imaging, an Inveon small animal PET scanner (Siemens) was used. Therefore, the animals were sacrificed 10 min or 60 min after injection of the radiolabeled compound and images were taken afterwards. The scintigraphic images were obtained using a γ-camera (Gamma Imager, Biospace, France). Cumulative images from 0–10 min, 60–70 min and 120–130 min after injection, were taken. For the biodistribution studies, the animals were sacrificed at certain time points, the organs were removed and weighed and the radioactivity of each sample was measured using a Cobra Auto γ-Counter, (Packard Bioscience, USA) in comparison with standards. The tissue-associated activity was related to the total injected dose (ID) and expressed as a percentage of the total injected dose per gram of tissue (%ID/g).

***In vivo* infection experiments**

Ethics statement
Animal studies were approved by the local government of Lower Franconia, Germany (approval number 55.2-2532-2-57) and performed in strict accordance with the guidelines for animal care and experimentation of German Animal Protection Law and the DIRECTIVE 2010/63/EU of the EU.
For the *in vivo* experiments, we applied *S. aureus* strain USA300 LAC* lux, which was constructed by transducing the luxABCDE operon from *S. aureus* Xen29 (PerkinElmer, Waltham, MA, USA) with phage φ11 into *S. aureus* LAC* (AH2163).[14] Overnight cultures of *S. aureus* USA300 LAC* lux in BHI medium were diluted to a final OD_{600} nm of 0.05 in 50 ml fresh BHI medium and grown for 3.5 hours at 37 °C. After centrifugation, the cell pellet was resuspended in BHI with 20 % glycerol, aliquoted and stored at -80 °C. For the generation of *in vivo* infection inocula, aliquots were thawed and washed twice with PBS. The desired bacterial concentration (5 x 10^7 CFU/100 µl) was then adjusted based on the respective OD_{600} nm values. A sample of the infection inoculum was plated on TSB agar plates in order to control the infection dose.
Female Balb/c mice (6 weeks, Janvier Labs, Le Genest-Saint-Isle, France) were used for all experiments.
Mice were infected by intravenous application of the bacterial inoculum via the tail vein. Treatment started at 2 hours p.i. and following doses were applied every 12 hours. The vancomycin group received 30 mg/kg body weight/ per day, the FU002 group received 56.4 mg/kg body weight/per day, the control group received equal amounts of sterile PBS at the respective time points. The mice were housed in individually ventilated cages under normal diet in groups of five throughout the experiment with ad libitum access to food and water. During infection, mice were scored twice daily and sacrificed at day 4 post infection and livers harvested. Organs were homogenized and plated in serial dilutions on TSB agar plates in order to determine colony-forming units (cfu). Significant differences in the CFU of the livers were determined with Kruskal-Wallis test (software: GraphPad Prism 5.0).

Results

Peptide synthesis

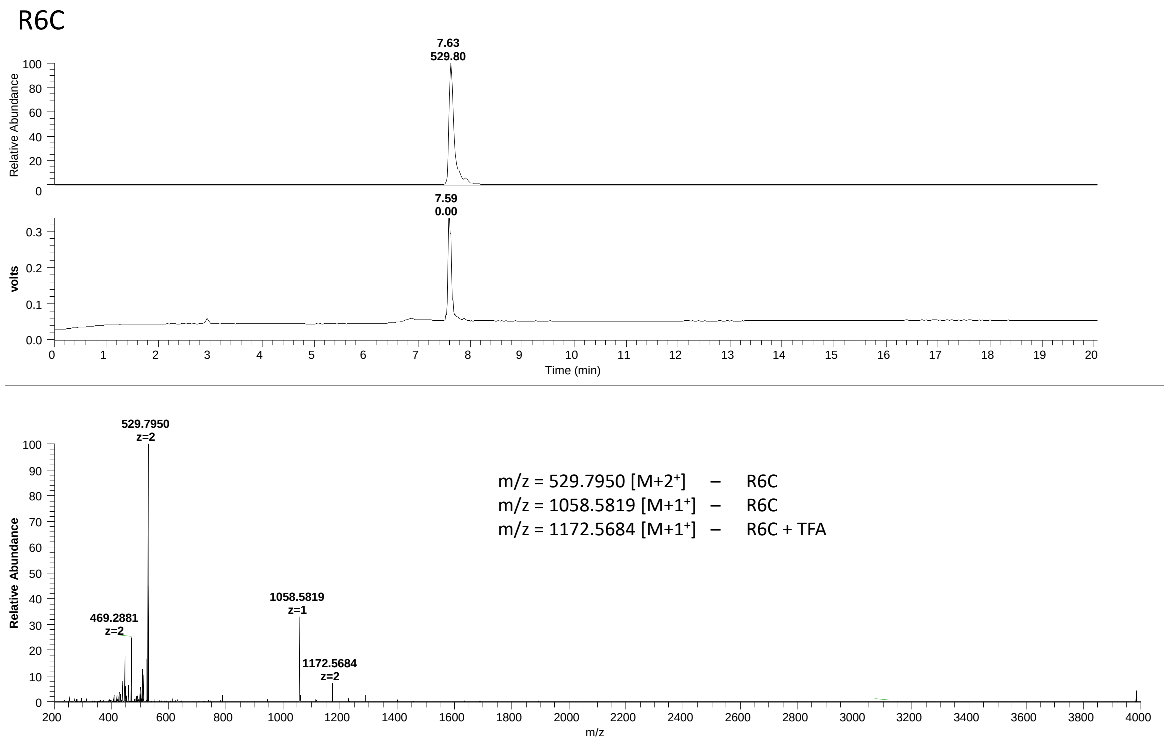

Figure S1 Mass spectrum of the peptide moiety of the lead candidate. Mass spectrum shows the peptide with a sufficient high purity for further procedure. Observed mass is m/z = 529.7950 [M+2$^+$], corresponding to the calculated mass of 1057.30 g/mol.

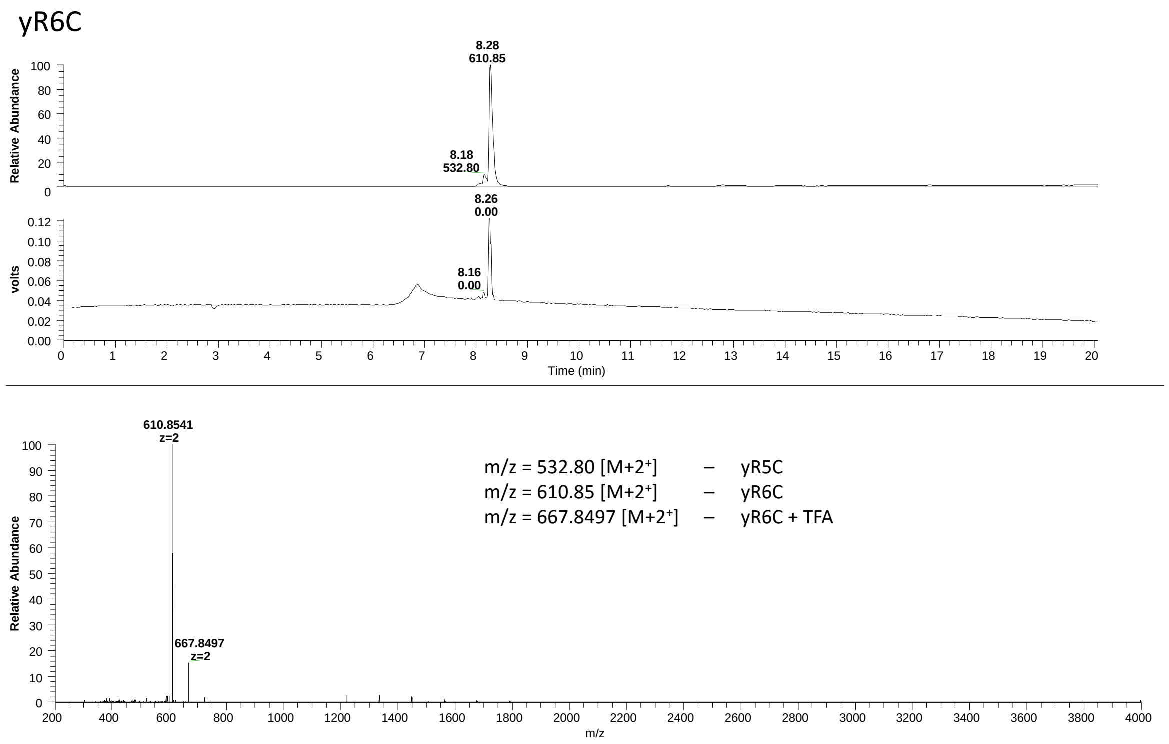

Figure S2 Mass spectrum of the peptide moiety of the lead candidate. Mass spectrum shows the peptide with a sufficient high purity for further procedure as the tyrosine derivative will be purified for the product again. Observed mass is m/z = 610.85 [M+2$^+$], corresponding to the calculated mass of 1220.48 g/mol.

Synthesis of lead compound FU002 (V_N-position)

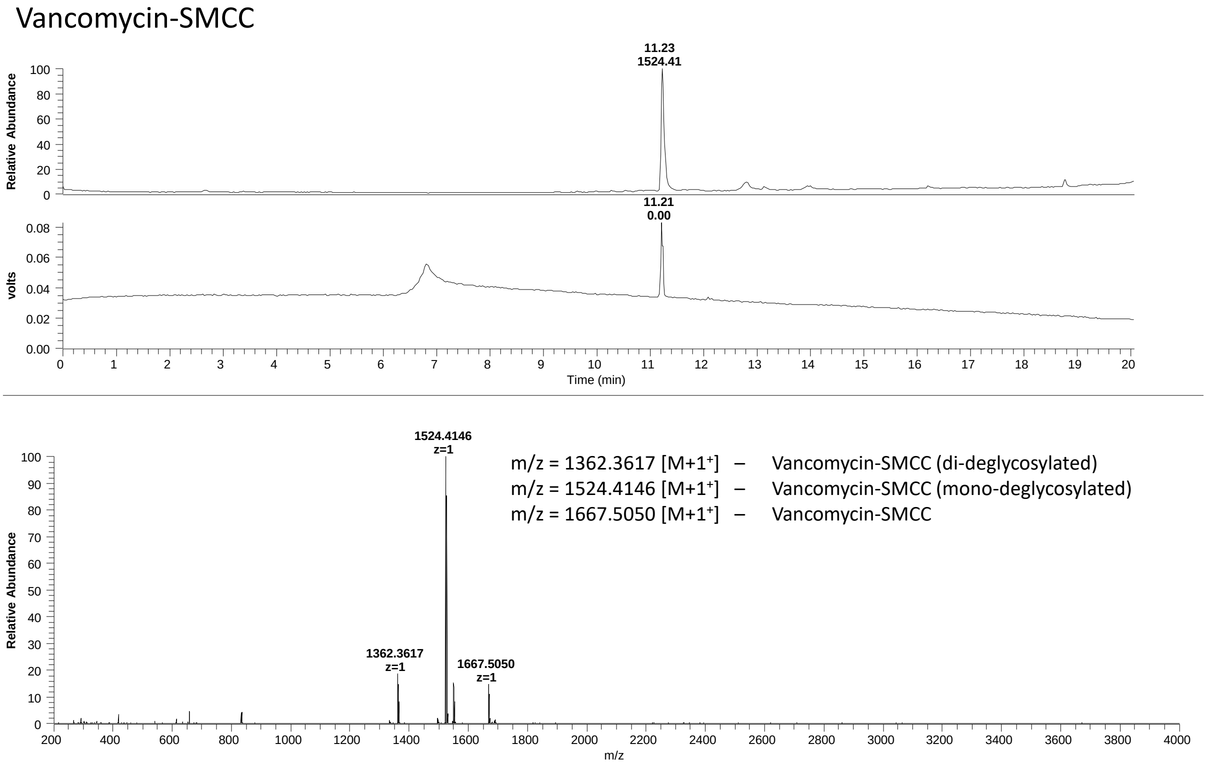

Figure S3 Mass spectrum of vancomycin-V_N-SMCC. Mass spectrum shows the conjugate. Mainly observed mass is m/z = 1524.4146 [$M+1^+$] due to ionization in mass spectrum the deglycosylated product is shown.

Table of synthesized compounds and corresponding MIC-values on *E. faecium* UL602570*

Table S1 The table shows the synthesized derivatives of vancomycin with their specific conjugation site, the used linker and peptide moiety and the corresponding minimal inhibitory concentration [mol/ml] on clinical isolate *E. faecium* UL602570*. All substances are tested in the described method on triplicates with a minimum of three independent experiments * = number of tests < 3; ** not tested in triplicates. AHX = ethylene diamine linked 6-maleimidohexanoic acid; eda = ethylene diamine

Substance	Site	Linker	Peptide moiety	Calculated molar mass [g/mol]	Minimal inhibitory concentration [mol/ml]
FU002	V_N	SMCC	R6C	2725.81	3.67×10^{-10}
FU005*	V_N	SMCC	R3C	2258.23	1.42×10^{-8}
FU006	V_N	SMCC	R1C	1945.85	$> 3.29 \times 10^{-8}$
FU007*	V_R	AHX	R6C	2771.88	4.07×10^{-9}
FU008	V_V	eda-SMCC	R6C	2768.88	4.23×10^{-10}
FU009	V_N	SMCC	KG6C	2260.15	$> 2.83 \times 10^{-8}$
FU013	V_C	eda-SMCC	R6C	2767.89	1.01×10^{-9}
FU021	V_N	SMCC	R9C	3194.38	5.32×10^{-10}
FU022	V_N	eda-SMCC	R9C	3237.45	1.07×10^{-9}
FU023	V_V	eda-SMCC	R9C	3237.45	2.69×10^{-10}
FU024	V_C	eda-SMCC	R9C	3236.46	5.25×10^{-10}
FU025	V_V	eda-SMCC	R3K3C	2684.84	1.01×10^{-9}
FU026	V_C	eda-SMCC	R3K3C	2683.85	2.01×10^{-9}
FU028**	V_N	SMCC	R3K3C	2641.77	2.00×10^{-9}
FU034	V_C	eda-SMCC	R3A3C	2512.56	4.35×10^{-9}
FU036*	V_N,V_V	eda-SMCC	R6C	4088.49	3.91×10^{-9}
FU043*	V_V,V_C	eda-SMCC	R6C	4087.51	9.79×10^{-10}
FU044*	V_N,V_C	eda-SMCC	R6C	4087.51	1.96×10^{-9}
FU049*	V_N	SMCC	W3R3C	2815.88	2.13×10^{-9}
FU052*	V_C	eda-SMCC	R3	2299.33	1.51×10^{-8}
FU067	V_N	SMCC	R3A3C	2470.48	3.12×10^{-8}
FU071*	V_V	eda-SMCC	R3	2300.31	1.89×10^{-9}
FU078*	V_N	SMCC	R3E3C	2644.59	$> 2.42 \times 10^{-8}$
FU079*	V_N	SMCC	(RE)3C	2644.59	$> 2.42 \times 10^{-8}$
FU080*	V_N	SMCC	E6C	2563.37	$> 2.50 \times 10^{-8}$
FU081*	V_N	SMCC	R3GPR3C	2879.98	1.39×10^{-9}
FU082*	V_N	SMCC	WR2LR3VRC	3124.32	2.56×10^{-9}
FU083*	V_N	SMCC	R2W2RW2C	3002.10	1.33×10^{-9}
FU084*	V_N	SMCC	KRW2KW2C	2946.07	1.36×10^{-9}
FU085*	V_N	SMCC	WK3RKVKC	2871.09	5.57×10^{-9}

Structure analysis of lead compound FU002 via deglycosylation

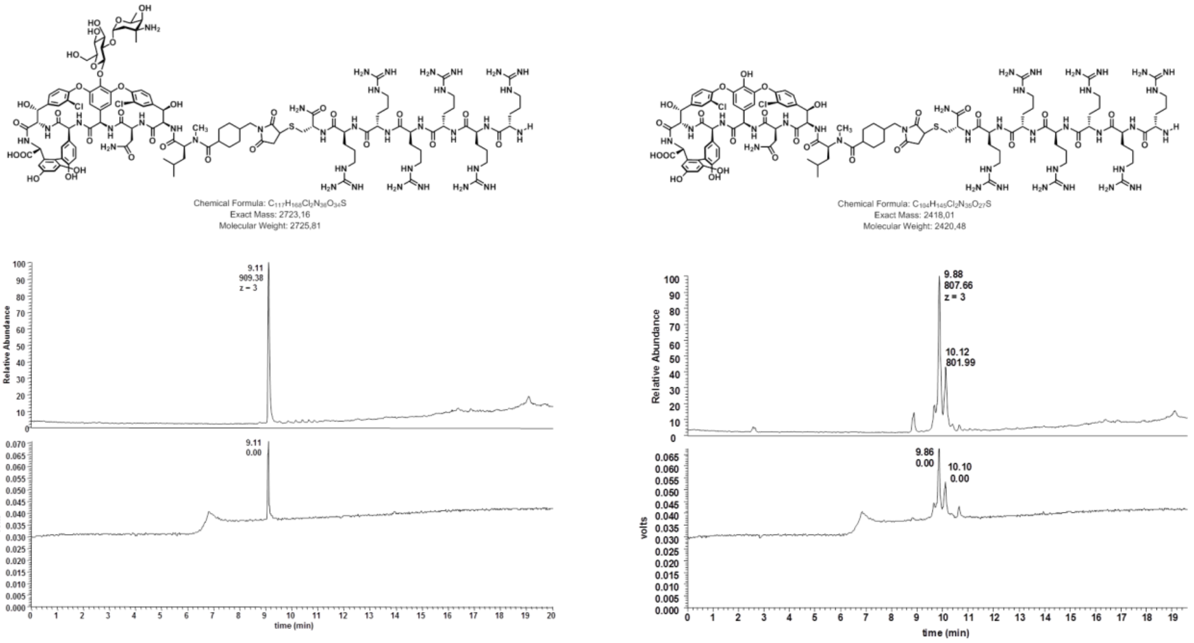

Figure S4 Mass spectrum of FU002 and its aglycon for verification of the coupling site. Mass spectrum shows the conjugate and the respective deglycosylated product.

Structure analysis of lead compound FU002 via NMR

NMR data

Legend: br, broad; d, doublet; m, multiplet; ol, overlapped; qt, quartet; t, triplet; s, singlet; v, very.

Vancomycin-SMCC

^{1}H (600 MHz, DMSO): 9.95, s; 9.38, s; 8.78, br d, J = 6.0 Hz; 8.70, v br d, J = 4.0 Hz; 7.82, br d, J = 1.3 Hz; 7.69, v br s; 7.62, 4H, v br s; 7.56, br dd, J = 8.4 and 1.3 Hz; 7.45, br dd, J = 8.4 and 1.3 Hz; 7.33, d, J = 8.4 Hz; 7.20, d, J = 8.4 Hz; 7.06, br s; 6.93, v br s; 6.90, 2H, s; 6.81, br dd, J = 8.4 and 1.8 Hz; 6.72, d, J = 8.6 Hz; 6.68, d, J = 2.2 Hz; 6.66, br s; 6.56, d, J = 2.3 Hz; 5.98, br d, J = 3.4 Hz; 5.94, d, J = 6.4 Hz; 5.82, d, J = 7.7 Hz; 5.67, s; 5.47, d, J = 6.6 Hz; 5.33, d, J = 5.8 Hz; 5.26, d, J = 7.8 Hz; 5.24, br d, J = 3.8 Hz; 5.16, br t, J = 3.5 Hz; 5.12–5.14, 2H, m; 5.07, d, J = 4.7 Hz; 4.82, v br d, J = 6.3 Hz; 4.67, qt, J = 6.5 Hz; 4.59, d, J = 6.3 Hz; 4.42, br d, J = 5.4 Hz; 4.15, br d, J = 11.7 Hz; 4.09, br s; 4.03, t, J = 5.4 Hz; 3.71–3.67, m; 3.56–3.51, 2H m; 3.43, ~qt, J = 7.8 Hz; 3.29–3.25, m; 3.18, br d, J = 6.5 Hz; 3.05, 2H, br d, J = 6.8 Hz; 2.72–2.62, br, m; 2.52, 3H, br s; 2.02–1.94, m; 1.93–1.85, m; 1.79–1.33, 8H, m; 1.29, 3H, s; 1.28–1.21, 2H, m; 1.06, 3H, d, J = 6.4 Hz; ca. 1.03, ol m; 0.93, 3H, d, J = 6.5 Hz; 0.87, 3H, d, J = 6.6 Hz; ca. 0.83, ol m; 0.74–0.62, 3H, m.

FU002

^{1}H (600 MHz, DMSO): 13.03, v br s; 10.02, s; 9.40, s; 9.14, v br s; 8.97, v br s; 8.79, br s; 8.68, br s; 8.59, br d, J = 6.9 Hz; 8.29–8.02, 10H, m; 7.87–7.77, 4H, m; 7.75, v br t,J = 5.6 Hz; 7.72–7.62, 4H, m; 7.57, br d, J = 7.8 Hz; 7.47, br d, J = 8.7 Hz; 7.34, d, J = 8.3 Hz; 7.26, br s; 7.19, br d, J = 8.2 Hz; 7.09, br s; 6.81, br d, J = 8.6 Hz; 6.77–6.67, 3H, m; 6.56, d, J = 2.2 Hz; 6.05–5.95, 2H, m; 5.81–5.77, m; 5.65, br s; 5.49–5.46, m; 5.37, br d, J = 5.4 Hz; 5.28–5.21, 2H, m; 5.20–5.08, 4H, m; 4.87, br s; 4.68, ~qt, J = 6.3 Hz; 4.57, br d, J = 5.9 Hz; 4.45, br s; 4.40–4.31, 2H, m; 4.29–4.11, 7H, m; 4.07–3.94, 3H, m; 3.82, br s; 3.68, br dd, J = 10.4 and 4.0 Hz; 3.57–3.50, 2H, m; 3.44, br qt, J = 7.3 Hz; 3.30–3.23, 2H, m; 3.22–3.02, 17H, m; 3.02–2.96, m; 2.61, 3H, v br s; 2.11–2.01, m; 1.94–1.86, m; 1.78–1.32, 36H, m; 1.29, 3H, s; 1.06, 3H, br d, J = 6.3 Hz; 1.02–0.95, 2H, m; 0.92, 3H, d, J = 6.3 Hz; 0.87, 3H, d, J = 6.2 Hz; 0.83–0.68, 2H, m.

Antimicrobial testing – Microdilution on non-resistant strain and *Cytotoxicity studies*

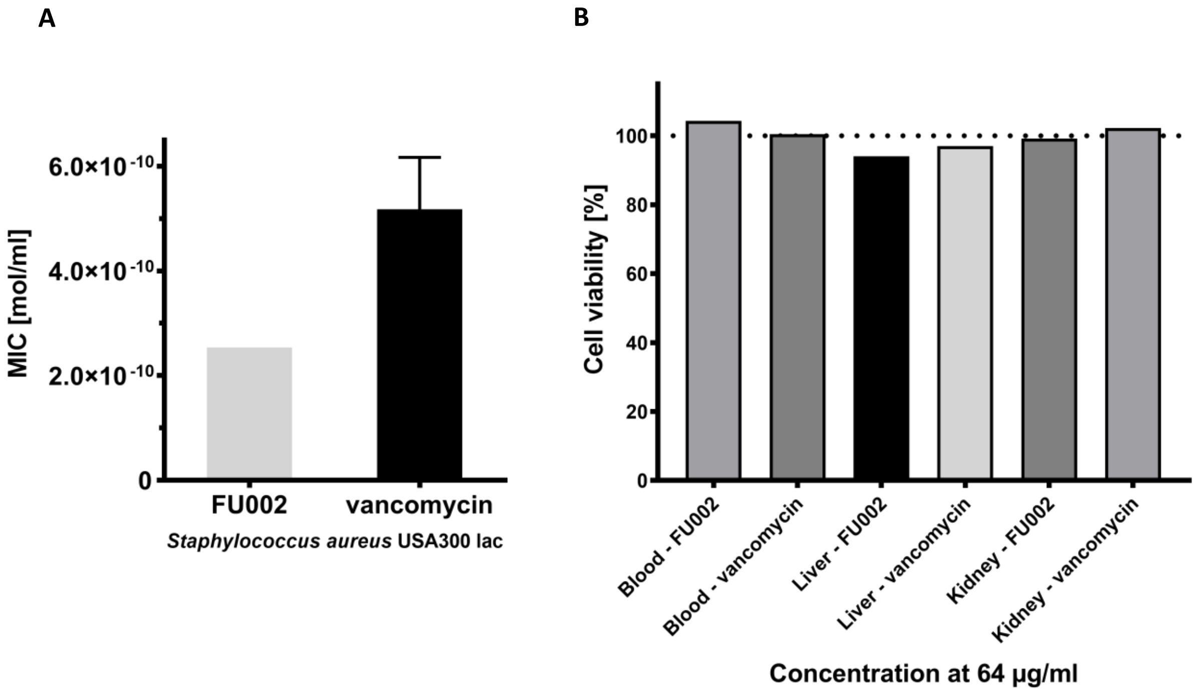

Figure S5 Minimal inhibitory concentration and cytotoxicity assay. (A) Comparison of FU002 and vancomycin in a microdilution assay on a vancomycin-sensitive strain *Staphylococcus aureus* USA300. FU002 is at least as active as vancomycin on a non-resistant strain. (B) Comparison of FU002 and vancomycin at a concentration of 64 µg/ml on three different human cell lines (blood, liver, kidney). FU002 shows no relevant cytotoxicity in this high concentration in comparison to its antimicrobial activity.

Antimicrobial testing – Microdilution and comparison

Table S2 MIC [µg/ml] of vancomycin and its derivative FU002 on Gram-negative bacterial strains. None of the tested substances shows relevant activity on tested Gram-negative strains.

Strain	MIC of FU002 [µg/ml]	MIC of vancomycin [µg/ml]
E. coli ATCC 25922	≥ 640	≥ 640
K. pneumoniae ATCC 700603	≥ 640	≥ 640
P. aeruginosa ATCC 27853	≥ 640	≥ 640
A. baumannii SC300007	≥ 640	≥ 640

Results are given as the median of at least triplicates on two different plates in the described set up for MIC testings.

Table S2 MIC [µg/ml] of vancomycin, FU002 and an equimolar mixture of the peptide and the unconjugated vancomycin. The non-covalently bound substance mixture shows no significant antimicrobial effect on E. faecium UL 602570*.

Substance	MIC [µg/ml] of *E. faecium* UL602570*
FU002	1
Equimolar mixture of R6C and vancomycin	> 64
vancomycin	> 64

Hemolysis

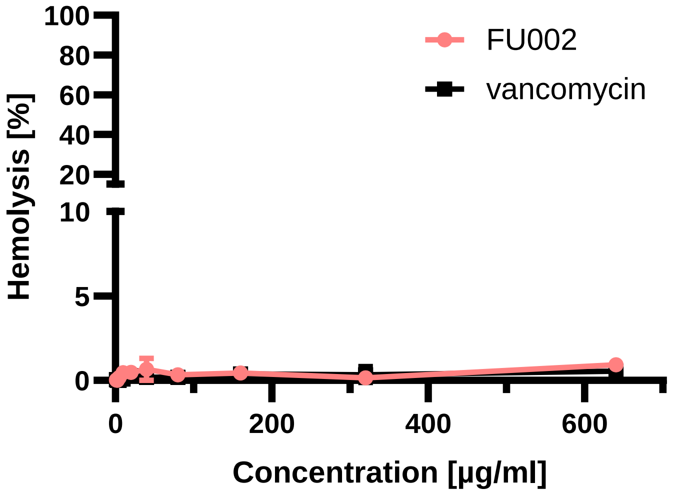

Figure S6 Hemolysis assay of vancomycin and its derivative FU002. Both substances show no relevant hemolytic activity up to a concentration of 640 µg/ml.

Biodistribution and pharmacokinetic studies

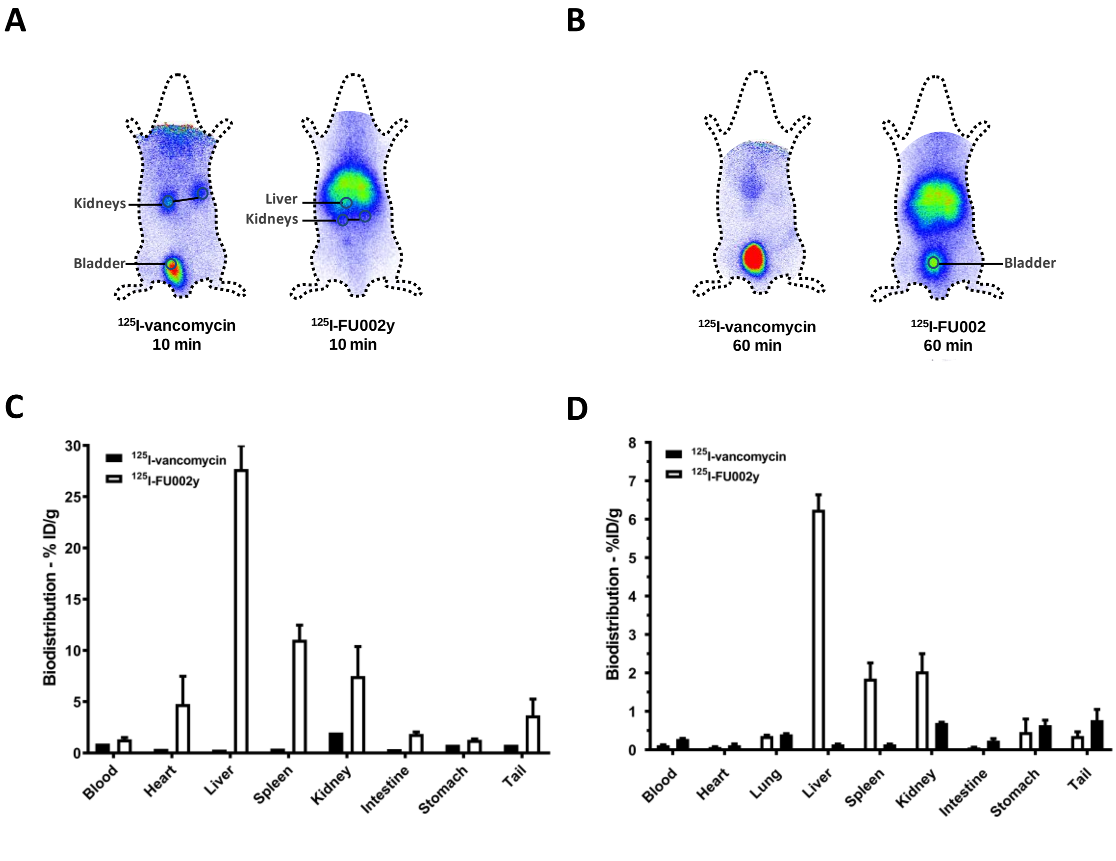

Figure S7 Molecular imaging of ^{125}I-vancomycin and its derivative ^{125}I-FU002y. (A) Scintigraphy 10 min post injection. Vancomycin shows a fast, renal excretion. In contrast, FU002 directs in the liver and the kidneys. (B) Scintigraphy 60 min post injection. Vancomycin is almost completely excreted and only found in the bladder. FU002 shows a much slower excretion. (C) The corresponding biodistribution of vancomycin and FU002 10 min post injection. FU002 shows a much higher organ uptake. (D) The biodistribution after 1 hour confirms the scintigraphic image. FU002 remains much longer in the corresponding organ.

Resistance development study

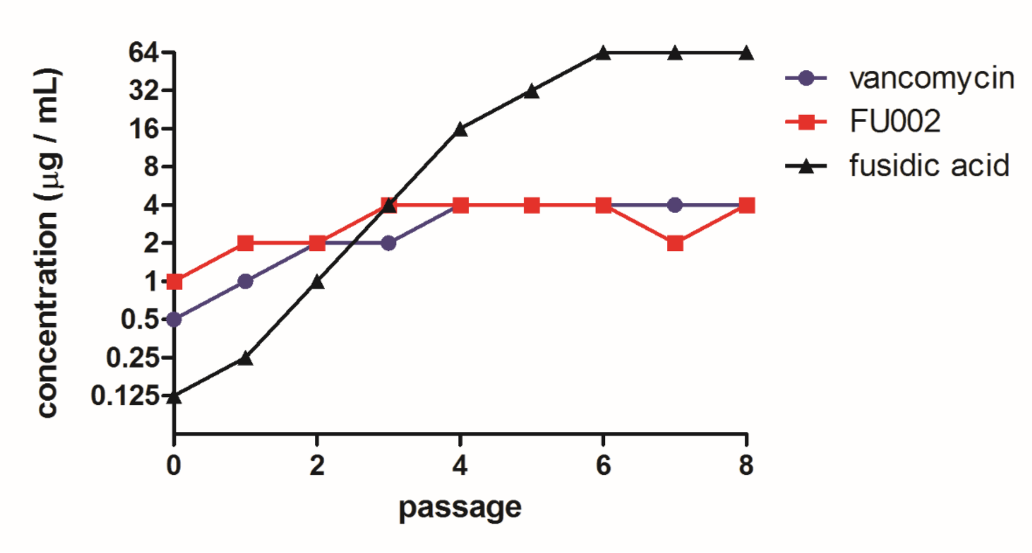

Figure S8 Resistance development studies of vancomycin, FU002 and fusidic acid on *Staphylococcus aureus* USA300 LAC. In these testings, the MRSA strain USA300 LAC showed an initial MIC value of 0.5 µg/ml and after 8 passages of 4 µg/ml for vancomycin. FU002 showed an initial MIC of 1 µg/ml and after 8 passages of 4 µg/ml. This minor increase in MIC values for both vancomycin and FU002 is usually observed in resistance development studies, it can be attributed to the adaption of the bacteria to the stress caused by the high concentration of the antibiotics. These results indicate the resilience of FU002 to resistance development, as shown by the fact that fusidic acid (known for fast resistance development) showed an increase of the MIC from 0.125 µg/ml to a MIC of 64 µg/ml within 8 passages.

References

1. Schieck, A. *et al.* Solid-phase synthesis of the lipopeptide Myr-HBVpreS/2-78, a hepatitis B virus entry inhibitor. *Molecules* **2010**, 15, 4773-4783.
2. Long, D.D. *et al.* A multivalent approach to drug discovery for novel antibiotics. *The Journal of Antibiotics* **2008**, 61, 595.
3. Klika, K.D. The Application of Simple and Easy to Implement Decoupling Pulse Scheme Combinations to Effect Decoupling of Large Values with Reduced Artifacts. *International Journal of Spectroscopy* **2014**.
4. Virta, P. *et al.* Synthesis, characterisation and theoretical calculations of 2, 6-diaminopurine etheno derivatives. *Organic & Biomolecular Chemistry* **2005**, 3, 2924-2929.
5. Balentova, E. *et al.* Stereochemistry, tautomerism, and reactions of acridinyl thiosemicarbazides in the synthesis of 1, 3-thiazolidines. *Journal of Heterocyclic Chemistry* **2006**, 43, 645-656.
6. Mäki, J., Tähtinen, P., Kronberg, L. & Klika, K.D. Restricted rotation/tautomeric equilibrium and determination of the site and extent of protonation in bi-imidazole nucleosides by multinuclear NMR and GIAO-DFT calculations. *Journal of Physical Organic Chemistry* **2005**, 18, 240-249.
7. Pearce, C.M. & Williams, D.H. Complete assignment of the 13C NMR spectrum of vancomycin. *Journal of the Chemical Society, Perkin Transactions* **1995***, 2*, 153-157.
8. CLSI. Methods for Dilution Antimicrobial Susceptibility Tests for Bacteria That Grow Aerobically; Approved Standard—Ninth Edition. CLSI document M07-A9. Wayne, PA: Clinical and Laboratory, Standards Institute; **2012**.
9. CLSI. Performance Standards for Antimicrobial Susceptibility Testing; Twenty-Fourth Informational Supplement. CLSI document M100-S24. Wayne, PA: Clinical and Laboratory Standards Institute; **2014**.
10. Determination of minimum inhibitory concentrations (MICs) of antibacterial agents by broth dilution
European Committee for Antimicrobial Susceptibility Testing (EUCAST) of the European Society of Clinical Microbiology and Infectious Diseases (ESCMID), EUCAST Discussion Document E. Dis 5.1 **2003**.
11. Domhan, C. *et al.* A novel tool against multiresistant bacterial pathogens: lipopeptide modification of the natural antimicrobial peptide ranalexin for enhanced antimicrobial activity and improved pharmacokinetics. *International Journal of Antimicrobial Agents* **2018**, 52, 52-62.
12. Uhl, P. *et al.* A liposomal formulation for the oral application of the investigational hepatitis B drug Myrcludex B. *European Journal of Pharmaceutics and Biopharmaceutics* **2016**, 103, 159-166.

13. Crim, J.W., Garczynski, S.F. & Brown, M.R. Approaches to radioiodination of insect neuropeptides. *Peptides* **2002**, 23, 2045-2051.

14. Boles B.R., Thoendel M., Roth A.J., Horswill A.R. Identification of genes involved in polysaccharide-independent Staphylococcus aureus biofilm formation. PLoS One **2010**, 5, e10146.
